# A Neutrophil Extracellular Traps–Related Signature Predicts Clinical Outcomes and Identifies Immune Landscape in Ovarian Cancer

**DOI:** 10.1111/jcmm.70302

**Published:** 2024-12-27

**Authors:** Yue Zhang, Chao Wang, Shanshan Cheng, Yanna Xu, Sijia Gu, Yaqian Zhao, Jiani Yang, Yu Wang

**Affiliations:** ^1^ Department of Gynecology, School of Medicine, Shanghai First Maternity and Infant Hospital Tongji University Shanghai China; ^2^ Shanghai Key Laboratory of Maternal Fetal Medicine, School of Medicine, Shanghai First Maternity and Infant Hospital, Shanghai Institute of Maternal‐Fetal Medicine and Gynecologic Oncology Tongji University Shanghai China; ^3^ Department of Obstetrics and Gynecology, School of Medicine, Renji Hospital Shanghai Jiaotong University Shanghai China

**Keywords:** neutrophil extracellular traps, ovarian cancer, prognostic signature, RAC2, tumour immune landscape

## Abstract

Ovarian cancer (OvCa) is the most lethal gynaecology malignancies worldwide. Neutrophil extracellular traps (NETs), net‐like protein structures produced by activated neutrophils and DNA‐histone complexes, have a central role in tumours, though haven't been fully explored in OvCa. We obtained transcriptome data from TCGA‐OvCa database (*n* = 376) as training, ICGC‐OvCa database (*n* = 111) as validation and GTEx database (*n* = 180) as controls. Through LASSO‐COX Regression analysis, we identified an eight‐gene signature among 87 NETs‐related genes, which was significantly related to poor prognosis in both TCGA‐OvCa and ICGC‐OvCa cohorts (Log‐rank *p*‐value = 0.0003 and 0.0014). Next, we constructed and validated a prognostic nomogram, consist of NETs‐related signature and clinical features (C‐index = 0.82). We evaluated 22 typical immune cell infiltration through CIBERSORT analysis, which implied upregulation of memory CD4 + T cells, follicular helper T cells and neutrophils in high‐risk group. Additionally, we predicted therapy sensitivity through TIDE algorithm, indicating that high NETs‐riskscore exhibited more sensitivity towards Sorafenib and less sensitivity towards immunotherapy. We initially reported that RAC2 upregulation was associated with NETs formation and poor prognosis (*p*‐value < 0.05) through IHC analysis of tissue microarrays (*n* = 125). Conclusively, NETs‐related signature was reliable for OvCa prognosis prediction and therapy assessment. Especially, RAC2 was predominantly related to NETs formation, thus providing hints towards anti‐tumour mechanism of NETs in OvCa.

## 1 Introduction

Ovarian cancer (OvCa) is the most lethal gynaecology malignancies worldwide, largely threatening women's health [1]. As reported, there were 19,680 new OvCa cases and 12,740 OvCa deaths, estimated for 2024 in the United States [2]. Unfortunately, nearly 70% OvCa patients were diagnosed at advanced clinical stages, with peripheral organs metastasis and invasion, which might result in 30% 5‐year overall survival rate (OS) [3]. Currently, OvCa treatment is standard surgery and platinum‐based chemotherapy, after which 70% patients would finally relapse [4]. Accordingly, in order to improve OvCa patients' prognosis in the realm of personalised treatment, further researches are needed to find biomarkers and explore corresponding mechanisms.

Neutrophil extracellular traps (NETs), net‐like protein structures produced by activated neutrophils and DNA‐histone complexes, have a central role in immune defence [5]. Emerging evidence indicates the role of NETs in cancer progression and metastasis, besides autoimmune diseases [6]. Recently, Yang et al. [7] implied that the DNA of NETs could enhance cancer metastasis via CCDC25, a protein that could activate the ILK‐β‐Parvin pathway to promote tumour cell motility in breast cancer. Deng et al. [8] reported that DDR1, a mediator of pro‐tumorigenic collagen signalling, could induce NET formation and drive pancreatic cancer metastasis. However, very few studies have reported the clinical application of NETs in OvCa and the underlying mechanism remains largely blank till now, partly due to its ‘cold’ immune microenvironment [9].

Here, we tried to define and validate a NETs‐related gene signature for prognosis prediction and drug sensitivity assessment among OvCa patients, based on bioinformatics analysis of public datasets. Additionally, we validated prognostic value of NETs‐related signature genes, especially RAC2, among OvCa patients through IHC analysis, PCR analysis and multiple immunofluorescence analysis, so as to assist decision‐making in OvCa.

## 2 Methods and Materials

### 2.1 Public Database Acquisition and Somatic Gene Mutation Analysis

We obtained RNA‐sequencing expression profile of ovarian tissues from TCGA dataset (https://portal.gdc.com; *n* = 376) as training, from ICGC dataset (https://dcc.icgc.org; *n* = 111) as validation and from GTEx dataset as normal controls (https://gtexportal.org; *n* = 180), up to October 2023. We then normalised gene RNA‐seq transcriptome profiles via the ‘limma’ R package. According to the Genecards database (https://www.genecards.org/), we identified potential NRGs (Relevance Score ≥ 20) by searching the term ‘Neutrophil Extracellular Traps’, ‘NETosis’ and ‘NETs’. The flowchart of this research is shown in Figure S1.

To identify functions related to the filtered differentially expressed NRGs (DE‐NRGs), we applied GO and KEGG enrichment analyses through the ‘ClusterProfiler’ R package. Next, we carried out protein–protein interaction (PPI) analysis through the Metascape software (https://metascape.org/), a database provides PPI network screens, so as to provide hints for protein interactions of DE‐NRGs.

### 2.2 Establishment and Estimation of the NETs‐Related Prognostic Signature

Firstly, we applied the LASSO Regression analysis to filter potential prognostic NRGs, with 10‐fold cross validation. To enhance model explicability, we conducted Cox Regression analysis to further distinguish riskscore formula of NETs‐related signature. According to the formula, we defined the NETs‐related riskscore of patients in TCGA‐OvCa cohort as training and ICGC‐OvCa cohort as validation, via the ‘glmnet’ R package. We then classified patients into two risk groups, based on median value as cut‐off. We carried out Cox Regression analysis to filter independent prognostic indicators via the ‘forestplot’ R package. Next, we developed a quantitative nomogram prognostic model and consist of the NETs‐related signature and clinical features, for OvCa patients' 1‐, 3‐ and 5‐year OS, via the ‘rms’ R package.

### 2.3 Single‐Cell Analysis of the NETs‐Related Signature

We downloaded the raw data of 10× Genomics single‐cell transcriptome expression from five OvCa patients and two normal controls, published by Qian et al. [10] in the GEO database. We removed poor‐quality genes (with < 3 detected cells) and poor‐quality cells (with < 200 detected genes). We preprocessed the raw data by calculating sequencing counts and analysing gene numbers via the ‘Seurat’ R package. We then evaluated the expression profile of cell‐types identified by specific markers. Next, we graphed pseudo‐time trajectory analysis to represent evolutionary association between cell‐types, including myeloid, epithelial and endothelial cells, during process of cell‐state transition via ‘monocle’ R package, through the TIGER (http://tiger.canceromics.org/) website [11].

### 2.4 Assessment of Tumour Immune Microenvironment Landscape and Drug Sensitivity

To visualise immune infiltration landscape, we conducted CIBERSORT analysis (https://cibersortx.stanford.edu/) to evaluate composition of 22 immune cells in tumour microenvironment. We estimated the gene expression profile of eight immune checkpoints, including LAG3, CTLA4, HAVCR2, CD274, PDCD1LG2, PDCD1, SIGLEC15 and TIGIT, between subgroups stratified via the NETs‐related signature. Based on the TIDE algorithm (http://tide.dfci.harvard.edu), we assessed patients' sensitivity towards immune checkpoint blockade (ICB) therapies. We then evaluated estimated IC50 values among OvCa cases for eight chemotherapies (Cisplatin, Bleomycin, Paclitaxel, Veliparib, Docetaxel, Vinblastine, Sorafenib and Gemcitabine), which were estimated based on the GDSC database (https://www.cancerrxgene.org), via the ‘pRRophetic’ R package.

### 2.5 Pan‐Cancer Analysis

To promote clinical application of NETs‐related signature, we carried out the pan‐cancer analysis based on RNA‐sequencing expression profile and corresponding clinical features of the TCGA pan‐cancer cohorts and normal controls, which were obtained from the UCSC Xena dateset (https://xena.ucsc.edu/). Those cancer types with less than 10 samples were excluded, so we finally involved 34 tumours in our pan‐cancer analysis. We normalised the gene RNA‐seq transcriptome profiles via the ‘limma’ R package. Next, we conducted Cox Regression analysis to distinguish prognostic value of the NETs‐related prognostic signature in the pan‐cancer cohorts. Then, we graphed the Kaplan–Meier (K–M) survival curves, which was compared by the Log‐rank test among risk groups stratified by the signature.

### 2.6 Immunohistochemistry Evaluation

We involved 125 OvCa patients at our institution from June 2007 to December 2013 and obtained 125 primary tumour samples and 40 metastatic tumour samples from them. The criteria for inclusion were as follows: (1) without prior or co‐existing cancers in 5 years; (2) histological‐confirmed OvCa; (3) obtained standard surgery and platinum‐based chemotherapy; and (4) had available follow‐up details and clinical information. Each patient was followed until May 2023. The research was approved by the Ethics Committee of the Renji Hospital Affiliated to Shanghai Jiaotong University School of Medicine, while informed consent from patients were obtained.

We hydrated and washed the samples, which was followed by the de‐waxed procedure of samples fixed in the formalin solution. Subsequently, we treated sections in the 3% H_2_O_2_ solution after the microwave antigen retrieval procedure to block endogenous peroxidase activity. We then incubated the slides overnight within Anti‐RAC2 Rabbit pAb (1:100, ABclonal, A1139) and subsequently within Mouse Anti‐Rabbit IgG (1:100, Sango Biotech, D110065). Two pathologists graded staining signal intensity for labelled cells in the slides via a four‐tier scale: 0 point as absent; 1 point as weak; 2 points as moderate; and 3 points as strong. They graded signal proportion for labelled cells in the slides via a four‐tier scale: 0 point as 0%–5%; 1 point as 6%–25%; 2 points as 26%–50%; 3 points as 51%–75%; and 4 points as > 75%. Next, according to the Immunoreactive score (IRS) formula: signal × proportion staining signal intensity, we analysed IRS score of each sample.

### 2.7 PCR Analysis

Refer to manufacturer instructions, total RNA of tissues was extracted via Trizol REAGENT (Servicebio, G3013). We reversely converted extracted RNA into cDNA via first Strand cDNA Synthesis Kit (Servicebio, G3330). Next, we conducted the PCR analysis using the SYBR Green PCR Mix (Bioss, C6028). The primer sequences were as follows: RAC2, Forward: 5′‐CAACGCCTTTCCCGGAGAG‐3′ and Reverse: 5′‐TCCGTCTGTGGATAGGAGAGC‐3′; SELL, Forward: 5′‐ACCCAGAGGGACTTATGGAAC‐3′ and Reverse: 5′‐GCAGAATCTTCTAGCCCTTTGC‐3′; GAPDH, Forward: 5′‐CTGGGCTACACTGAGCACC‐3′ and Reverse: 5′‐AAGTGGTCGTTGAGGGCAATG‐3′. We then set GAPDH as the internal control primer and analysed comparative mRNA expression using the 2^−ΔΔCt^ method.

### 2.8 Statistics Analysis

In our study, we applied the chi‐square test to analyse categorical variables and the *t*‐test to analyse continuous variables. To address potential false positives, we have applied Bonferroni corrections. We visualised the K–M survival curves and evaluated them by the Log‐rank test. In addition, receiver operating characteristics (ROC) curves were graphed and then evaluated by the area under the curve (AUC) value. To filter independent prognostic indicators, we applied univariate Cox Regression models to evaluated the prognostic value of the defined NETs‐related signature, as well as conventional clinical characteristics reported by previous studies, such as age, tumour size, tumour side, grade and clinical FIGO stage. Then, we included those indicators with significant value filtered by the univariate Cox Regression analysis (*p*‐value < 0.05) into the multivariate model. Before the Cox Regression analysis, we check the proportional hazards assumption through cumulative risk function curves. For those covariates don't fit the proportional hazards assumption, we applied the Time‐Dependent Cox Regression Model. We applied all statistical analyses through the R software (Version 4.0.3), while *p*‐value < 0.05 was defined as statistical‐significant.

## 3 Results

### 3.1 Identification of Differentially Expressed NETs‐Related Genes in OvCa

**FIGURE 1:**
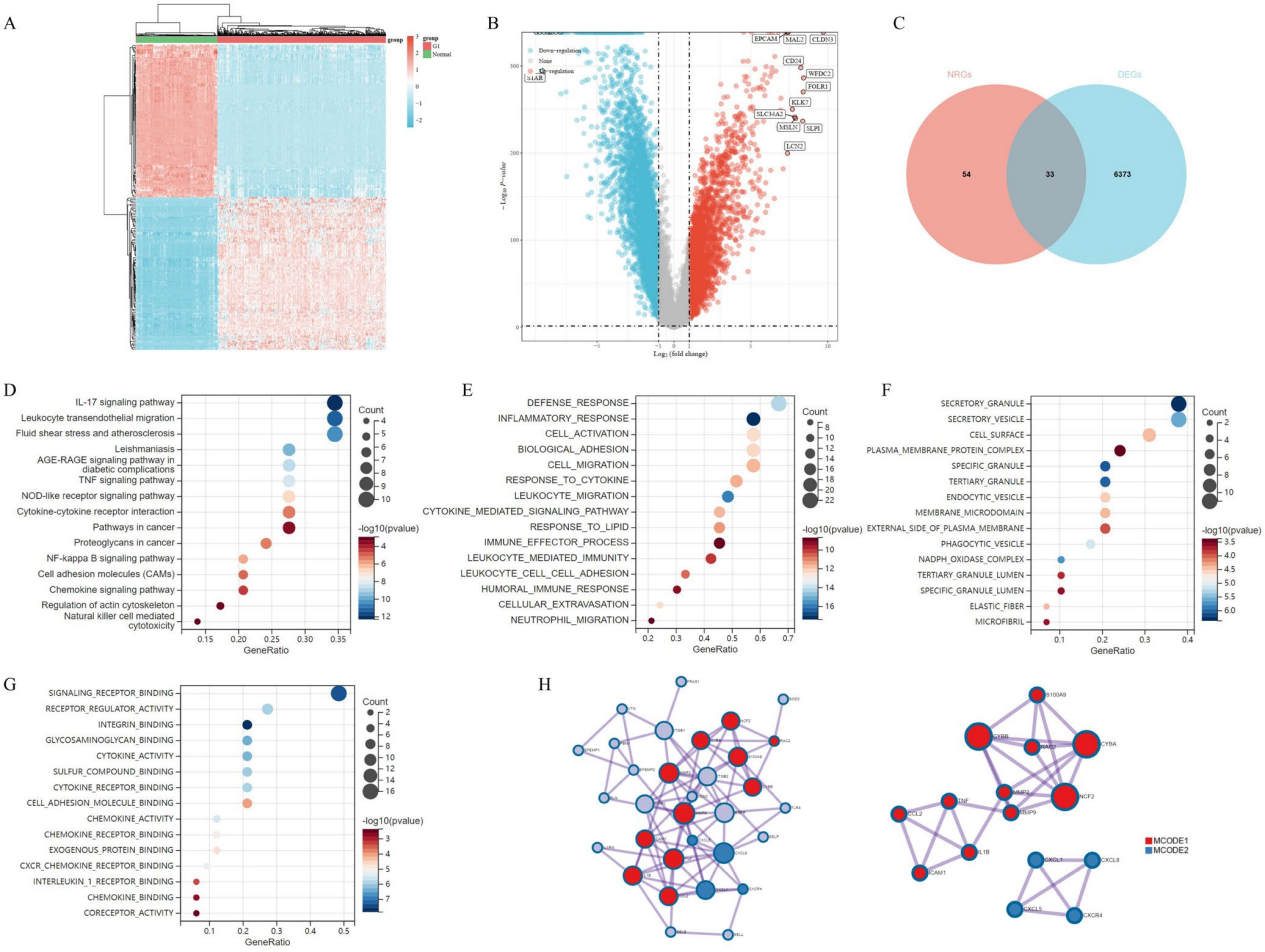
Identification of differentially expressed NETs‐related genes (NRGs) in OvCa. (A) Heatmap diagram showed the expression profile of ovarian tissues from TCGA‐OvCa cohorts and GTEx normal controls. (B) Volcano plot of differentially expressed genes between OvCa and normal tissues. (C) Venn diagram identified 33 differentially expressed NRGs (DE‐NRGs). (D) Top 15 KEGG pathway enrichment analysis of 33 DE‐NRGs. The circle size represents gene ratio, and circle colour implies *p*‐value. Top 15 GO pathway enrichment analyses of 33 DE‐NRGs refer to (E) biological process, (F) cellular component and (G) molecular function. (H) The protein–protein interaction network plot of 33 DE‐ARGs (left), while the hub genes were highlighted in red and blue (right).

We obtained transcriptome rawdata of OvCa patients (*n* = 376) from the TCGA database, and the expression profile of normal controls (*n* = 180) from GTEx database (Figure 1A). The volcano diagram identified 6406 DEGs between TCGA‐ OvCa tissues and normal controls, among which 2333 DEGs were upregulated and 4073 DEGs were down‐regulated in OvCa (Figure 1B). We obtained 87 NRGs (Relevance Score ≥ 20) by searching the term ‘Neutrophil Extracellular Traps’, ‘NETosis’ and ‘NETs’ on the Genecards dataset (https://www.genecards.org/). In Figure 1C, we defined 33 genes as DE‐NRGs, NRGs which were differentially expressed between OvCa and normal controls. Then, we carried out GO and KEGG pathway enrichment analysis of the 33 potential DE‐NRGs. The top 15 KEGG pathways were mainly enriched in Leukocyte transendothelial migration, IL‐17 signalling pathways and pathways in cancer, etc. (Figure 1D). The top 15 GO pathway enrichment analyses of 33 DE‐NRGs refer to biological process, cellular component and molecular function (Figure 1E–G, respectively) were mainly enriched in defence response, secretory granule and signalling receptor binding, etc. We applied PPI network analysis of 33 DE‐NRGs (left) to hint protein interactions, while hub genes were highlighted in red and blue (right), based on Metascape website (https://metascape.org) (Figure 1H) [12].

### 3.2 Establishment and Estimation of the NETs‐Related Prognostic Signature

**FIGURE 2:**
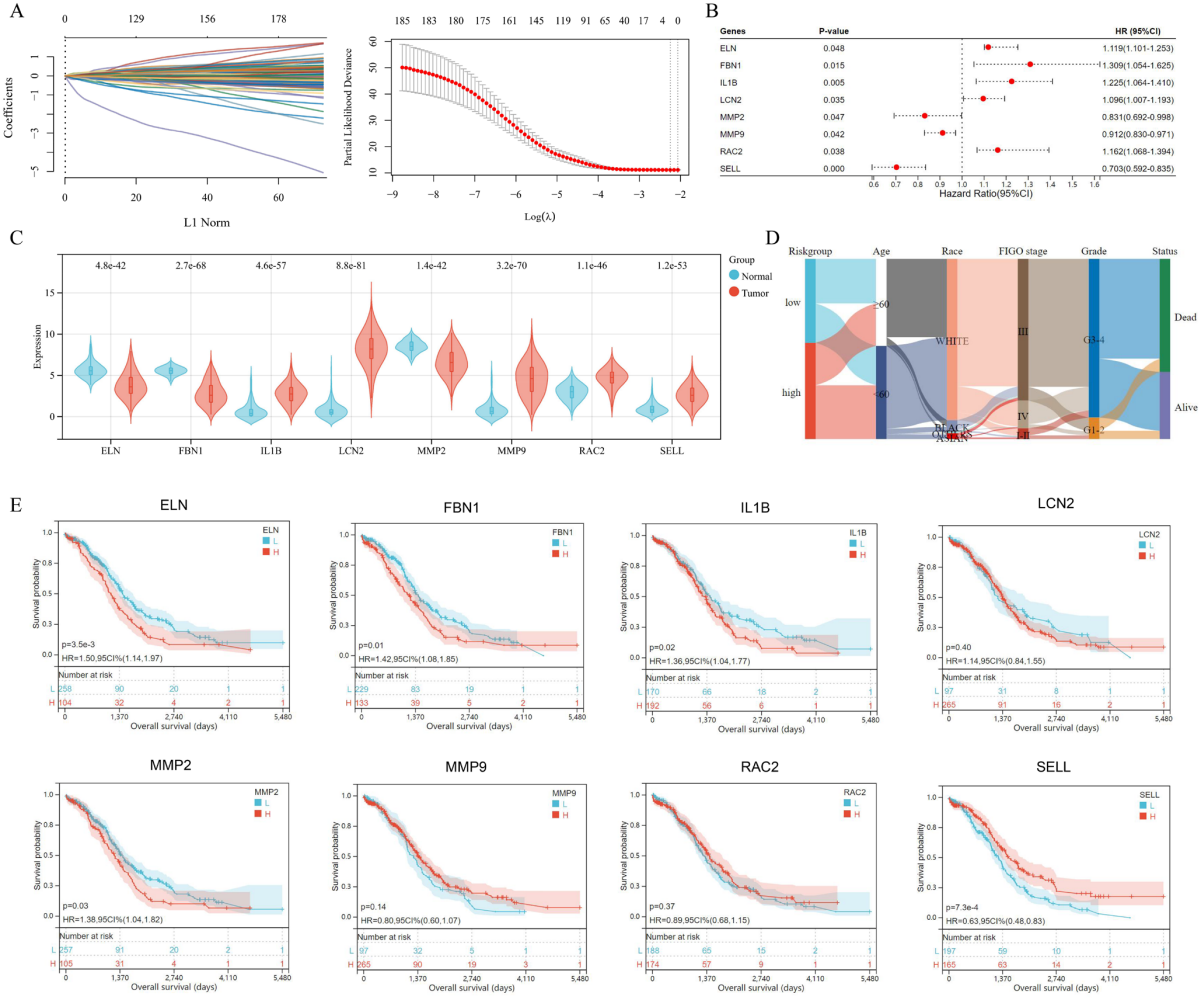
Establishment of NETs‐related prognostic signature in OvCa. (A) λ selection plot of LASSO parameter selection. (B) Forest diagram indicated prognostic ability of eight prognostic NETs‐related genes (NRGs), including RAC2, IL1B, MMP9, LCN2, MMP2, ELN, SELL and FBN1, which were evaluated by the LASSO‐Cox analysis. (C) Expression profile of eight prognostic NRGs in OvCa and control tissues. (D) Sankey diagram of NETs‐related signature and clinical characteristics (age, race, grade, clinical stage and survival status). (E) Kaplan–Meier curves for overall survival rate refer to the eight prognostic NRGs, including ELN, FBN1, IL1B, LCN2, MMP2, MMP9, RAC2 and SELL, respectively.

Through LASSO analysis, we identified eight potential prognostic NRGs (ELN, FBN1, IL1B, LCN2, MMP2, MMP9, RAC2 and SELL) from the 33 DE‐NRGs (Figure 2A). To enhance the model explicability, we carried out Cox Regression analysis to further distinguish a NETs‐related eight‐gene prognostic signature (Figure 2B) as follows: Riskscore = (0.156) × RAC2 + (0.2087) × IL1B + (−0.0993) × MMP9 + (0.0961) × LCN2 + (−0.1773) × MMP2 + (0.1161) × ELN + (−0.3714) × SELL + (0.2679) × FBN1. Table S1 showed a brief view of the eight identified NETs‐related signature genes [13, 14, 15, 16, 17, 18, 19, 20, 21, 22]. Figure 2C showed the expression profile of eight prognostic signature genes in OvCa and normal tissues. The Sankey plot (Figure 2D) visualised the relationship between the NETs‐related signature and clinical characteristics, including age, race, clinical FIGO stage, grade and survival status. In Figure 2E, the K–M survival curves for OS rate refer to eight prognostic NRGs indicated that OvCa patients with high‐expressed ELN, FBN1, IL1B and MMP2 suffered worse OS, while those with high‐expressed SELL had better prognosis (*p*‐value < 0.05). However, K–M curves of LCN2, RAC2 and MMP9 showed no significance (*p*‐value ≥ 0.05), which should be validated in other datasets.

According to above formula, we estimated NETs‐related riskscore of patients in the TCGA‐OvCa cohort (*n* = 376) as training and ICGC‐OvCa (*n* = 111) cohort as validation. We divided patients into two subgroups, based on median value as cut‐off. In the TCGA‐OvCa (Figure S2A) and ICGC‐OvCa cohorts (Figure S2B), the distribution (top) and scatter diagrams (middle) showed NETs‐related riskscore of each OvCa patient, refer to survival time and status, while the heatmaps (bottom) represented the expression of eight NETs‐related signature genes, including ELN, FBN1, IL1B, LCN2, MMP2, MMP9, RAC2 and SELL, between two risk groups. Through K‐M survival curves, we concluded that high‐risk OvCa patients had worse OS in both TCGA‐OvCa and ICGC‐OvCa cohorts (*p*‐value = 0.0003 and 0.0014, Figure S2C,D). The ROC analysis showed that NETs‐related eight‐gene signature had promising prediction values for OS at 5‐year interval in the TCGA‐OvCa cohort and at 1‐ and 3‐year intervals in the ICGC‐OvCa cohort (AUC value > 0.65, Figure S2E,F). However, the AUC value for 1‐ and 3‐year OS of TCGA‐OvCa cohort and 5‐year OS of ICGC‐OvCa cohort is relatively low (AUC value < 0.65).

### 3.3 Construction and Validation of the NETs‐Related Nomogram

**FIGURE 3:**
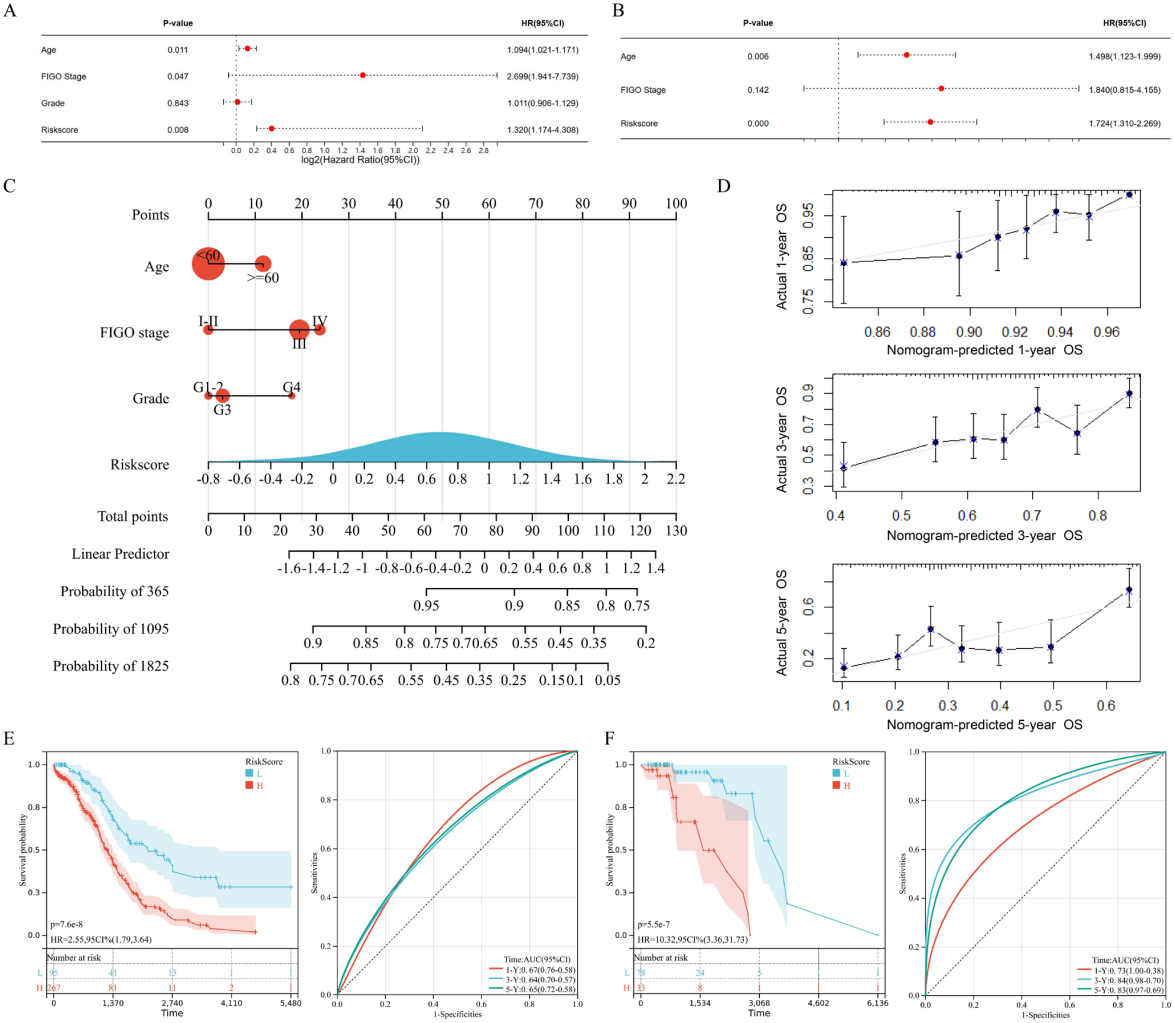
Construction and validation of NETs‐related nomogram for OvCa patients. Forest diagrams for (A) univariate and (B) multivariate Cox Regression analysis of NETs‐related signature riskscore and clinical features, such as age, clinical stage and pathological grade. (C) Nomogram prognostic model for OvCa patients' 1‐, 3‐ and 5‐year OS, according to NETs‐related signature and clinical features. (D) Calibration diagrams of NETs‐related nomogram to predict 1‐year (top), 3‐year (middle) and 5‐year OS (bottom). Kaplan–Meier survival curves for OvCa individuals divided by NETs‐related nomogram score, in the (E) TCGA‐OvCa training and (F) ICGC‐OvCa validation cohorts.

The correlation between NETs‐related riskscore and clinical features of TCGA‐OvCa patients were listed in Table S2, which indicated that the covariates were not significantly correlated (*p*‐value > 0.01). Next, based on NETs‐related signature riskscore and clinical indicators, including age, pathological grade and clinical stage, we carried out univariate (Figure 3A) and multivariate (Figure 3B) Cox Regression analysis to identify prognostic factors among OvCa patients. We checked the proportional hazards assumption through cumulative risk function curves, which indicated that grade did not fit the proportional hazards assumption (Figure S3A–D). So, we then applied the Time‐Dependent Cox Regression Model, which showed that NETs‐related riskscore (*p*‐value = 0.000) and age (*p*‐value = 0.006) were prognostic factors. The results showed that OvCa patients with high NETs‐related riskscore were associated with a 1.724‐fold increase (95% CI 1.310–2.269) of death rate and advanced age were associated with a 1.489‐fold increase (95% CI 1.123–1.999) of death. Next, in Figure 3C, we developed a quantitative nomogram prognostic model for OvCa patients' 1‐, 3‐ and 5‐year OS, according to the NETs‐related signature and clinical features, with a C‐index of 0.82. In Figure 3D, calibration plots of the NETs‐related nomogram visualised ideal consistency between observed and predicted 1‐year (top), 3‐year (middle) and 5‐year OS (bottom). In addition, we validated optimum performance of nomogram model in the TCGA‐OvCa cohort (Figure 3E) and the ICGC‐OvCa cohort (Figure 3F), through K–M curve analysis (left, *p*‐value < 0.001) and ROC analysis (right, AUC value > 0.65).

### 3.4 Single‐Cell Analysis and Somatic Alteration Landscape of the NETs Pattern

We downloaded the raw data of 10× Genomics single‐cell transcriptome expression from five OvCa patients and two normal controls, published by Qian et al. [10] in the GEO database. We then evaluated the expression profile of 10 cell‐types identified by specific markers (Figure S4A). Next, we graphed pseudo‐time trajectory analysis to represent evolutionary association between cell‐types, during process of cell‐state transition (Figure S4B). Especially, trajectory inference revealed evolutionary pathways of Myeloid cells from OvCa lesions, with (Figure S4C) RAC2 and (Figure S4D) SELL expression overlapped along pseudo‐time. Accordingly, in Figure S4E, we conducted pseudo‐time trajectory analysis in OvCa tissues of Myeloid cells, along with (Figure S4F) RAC2 and (Figure S4G) SELL expression. To reveal genomic aberrations landscape of according to the NETs‐related signature, we analysed mutation frequency and somatic‐cell copy number alternation of TCGA‐ OvCa patients, divided by the NETs‐related signature. Figure S4H indicated genes with highest mutation frequency as TP53 (92.5%), TTN (37.3%) and CSMD3 (14.1%).

### 3.5 Immune Microenvironment Landscape and the NETs‐Related Signature

**FIGURE 4:**
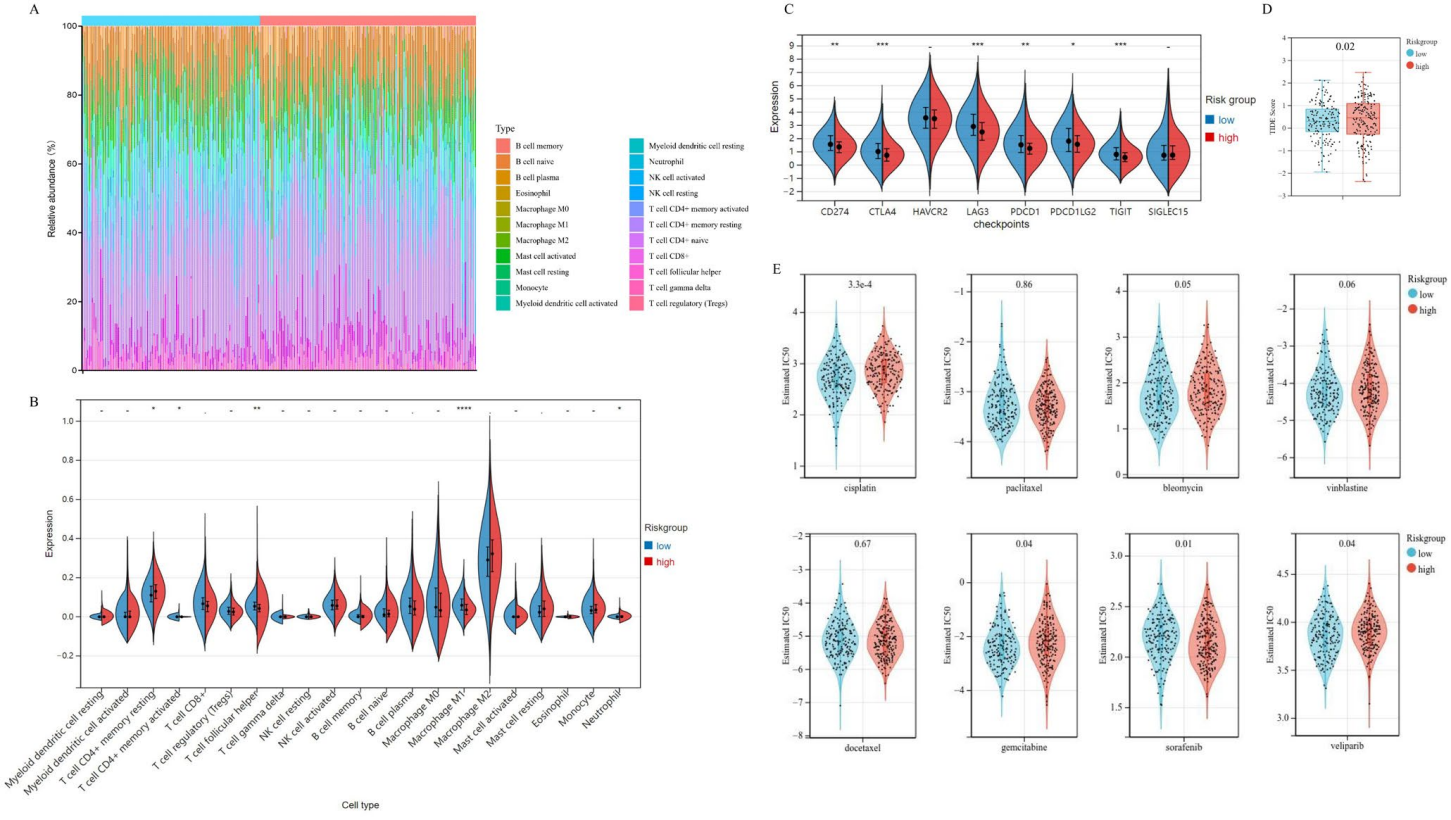
Analysis for tumour immune microenvironment landscape and immunotherapy/chemotherapy sensitivity related to the NETs‐related signature. (A) Boxplots represented composition of 22 immune cells infiltrating in OvCa tissues of TCGA‐OvCa patients, who were then stratified into two risk groups referring to the NETs‐related signature. (B) Violin diagrams showed expression profile of 21 typical immune cells infiltration in two risk groups. (C) Boxplots represented gene expression profile of eight typical immune checkpoints, LAG3, CD274, CTLA4, HAVCR2, PDCD1LG2, PDCD1, SIGLEC15 and TIGIT, between two NETs‐related risk groups. (D) Assessment of sensitivity towards immune checkpoint blockade therapies refers to the Tumour Immune Dysfunction and Exclusion (TIDE) score. (E) The violin plots indicated estimated IC50 values among OvCa individuals, as for eight chemotherapies, which were estimated based on the GDSC database. *p‐value < 0.05; **p‐value < 0.01; ***p‐value < 0.001; ****p‐value < 0.0001.

Emerging evidence supported cross‐talk of tumour cells and immune cells in OvCa microenvironment, which might contribute to tumour metastasis [23]. We then conducted the CIBERSORT analysis to evaluate composition of 22 immune cells infiltrating in OvCa tissues of TCGA‐OvCa patients, who were then stratified into two risk groups referring to the NETs‐related signature (Figure 4A). However, considering that the concentration of CD4+ naïve T cell was extremely low in the involved OvCa tissue, we only graphed expression profile of 21 typical immune cells (except for CD4+ naïve T cell) infiltration in two risk groups (Figure 4B). The violin diagrams indicated that follicular helper T cells, resting memory CD4+ T cells and neutrophil were significantly upregulated in high‐risk group, while M1 macrophage cells and activated memory CD4+ T cells were upregulated in low‐risk group. Next, we applied correlation analysis of 22 typical immune cells, which indicated positive relationship between Regulatory T cells (Tregs) and M2 macrophage cells. Except for intense relationship between resting and activated cells, we also found a negative relationship between the Macrophage M1 cells and CD4+ naive T cells (*p*‐value < 0.001, Figure S5).

### 3.6 Immunotherapy and Chemotherapy Sensitivity Associated With the NETs‐Related Signature

In addition, we estimated the expression profile of eight immune checkpoints, such as LAG3, CD274, CTLA4, HAVCR2, PDCD1LG2, PDCD1, SIGLEC15 and TIGIT, between two NETs‐related risk groups. The result showed that LAG3, CTLA4, CD274, PDCD1, PDCD1LG2 and TIGIT were upregulated in low‐risk group (Figure 4C, *p*‐value < 0.05). High‐risk OvCa patients were less likely to benefit from immunotherapies of these six checkpoints. Based on the TIDE algorithm, we assessed patients' sensitivity towards ICB therapies, which showed that high‐risk individuals also had higher TIDE scores, representing poor prognosis after the ICB immunotherapies (Figure 4D, *p*‐value = 0.02).

We then evaluated estimated IC50 values among OvCa individuals for eight chemotherapies (Cisplatin, Bleomycin, Paclitaxel, Veliparib, Docetaxel, Vinblastine, Sorafenib and Gemcitabine), which were estimated based on the GDSC database. Figure 4E showed that the estimated IC50 for Cisplatin, Gemcitabine and Veliparib were significantly higher among high‐risk individuals, while IC50 for Sorafenib was lower (*p*‐value < 0.05).

### 3.7 Pan‐Cancer Analysis of NETs‐Related Signature

**FIGURE 5:**
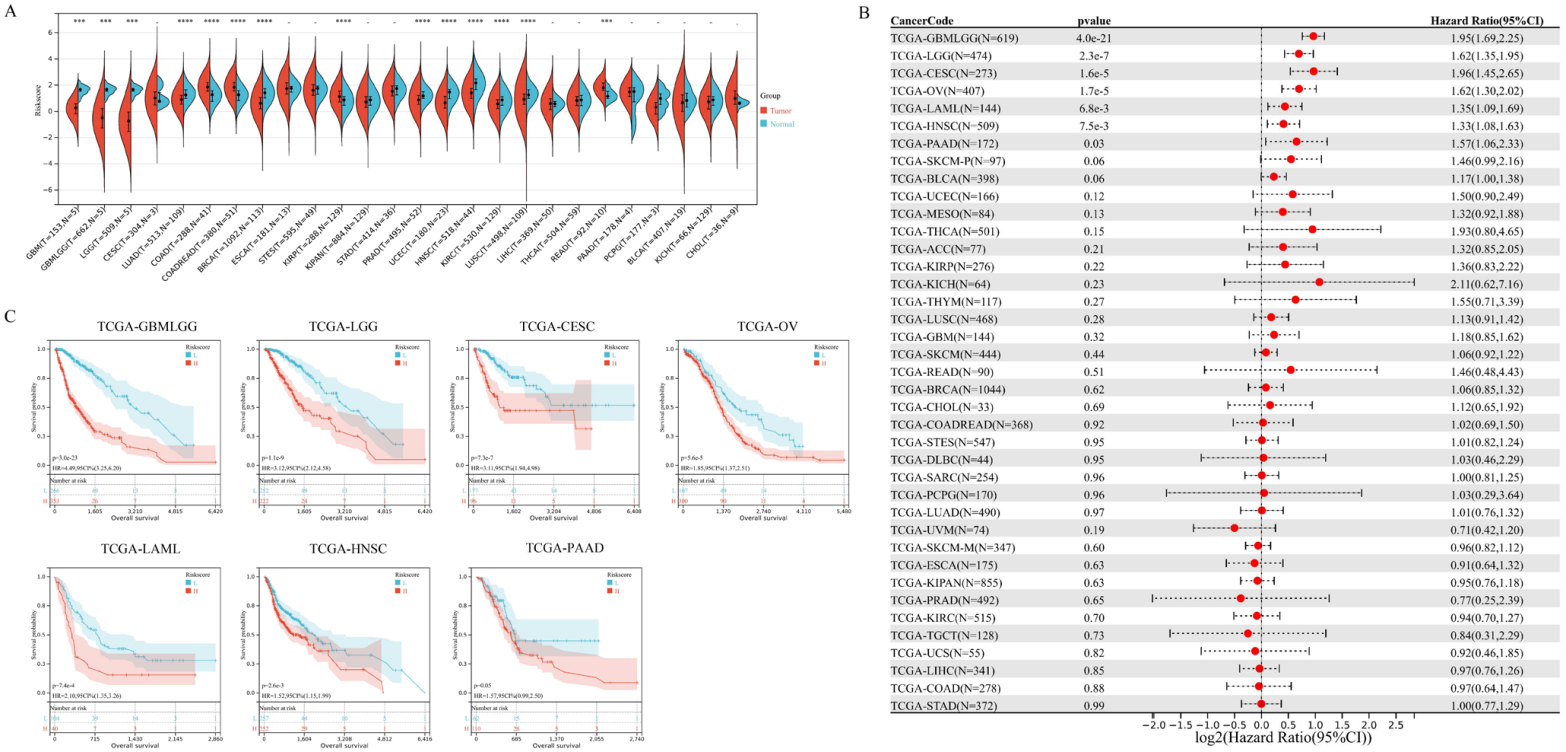
Pan‐cancer analysis of the NETs‐related signature. (A) Violin chart represented NETs‐related signature riskscore distribution of tumour tissues in pan‐cancer and controls. (B) The forest chart distinguished prognostic value of the signature via the Cox Regression algorithm in pan‐cancer. (C) The Kaplan–Meier curves of Glioma, Cervical squamous cell carcinoma and endocervical adenocarcinoma (CESC), Low‐grade glioma (LGG), Acute Myeloid Leukaemia (LAML), OvCa, Head and Neck squamous cell carcinoma (HNSC) and Pancreatic adenocarcinoma (PAAD) in the TCGA cohorts, which were classified by NETs‐related signature.

To promote clinical application of NETs‐related signature, we carried out the pan‐cancer analysis based on the TCGA pan‐cancer dataset including 34 tumours. We analysed gene expression of RAC2 and SELL in Figure S6A,B, which showed that Non‐small cell lung carcinoma (NSCLC) ranked the highest RAC2 and SELL expression. We compared the NETs‐related signature riskscore distribution of tumour tissues in pan‐cancer and corresponding controls, which showed that almost all malignancies had significantly different riskscores, compared with normal controls (Figure 5A). Through the Cox Regression method, we distinguished prognostic value of the NETs‐related prognostic signature in the TCGA cohorts (Figure 5B). The K‐M curves validated optimum performance (*p*‐value < 0.05) of the NETs‐related signature among Glioma, Cervical squamous cell carcinoma and endocervical adenocarcinoma (CESC), Low‐grade glioma (LGG), Acute Myeloid Leukaemia (LAML), OvCa, Head and Neck squamous cell carcinoma (HNSC) and Pancreatic adenocarcinoma (PAAD) in the TCGA cohorts (Figure 5C). Moreover, we explored association between the immune landscape and NETs‐related riskscore in pan‐cancer, which was analysed via CIBERSORT algorithm (Figure S6C). The findings indicated that M2 macrophage and memory CD4+ T cells were positively associated with the NETs‐related pattern among pan‐cancer, while Treg and memory B cells were inversely associated.

### 3.8 Aberrant Downregulation of RAC2 Related to OvCa Metastasis and Poor Prognosis

**FIGURE 6:**
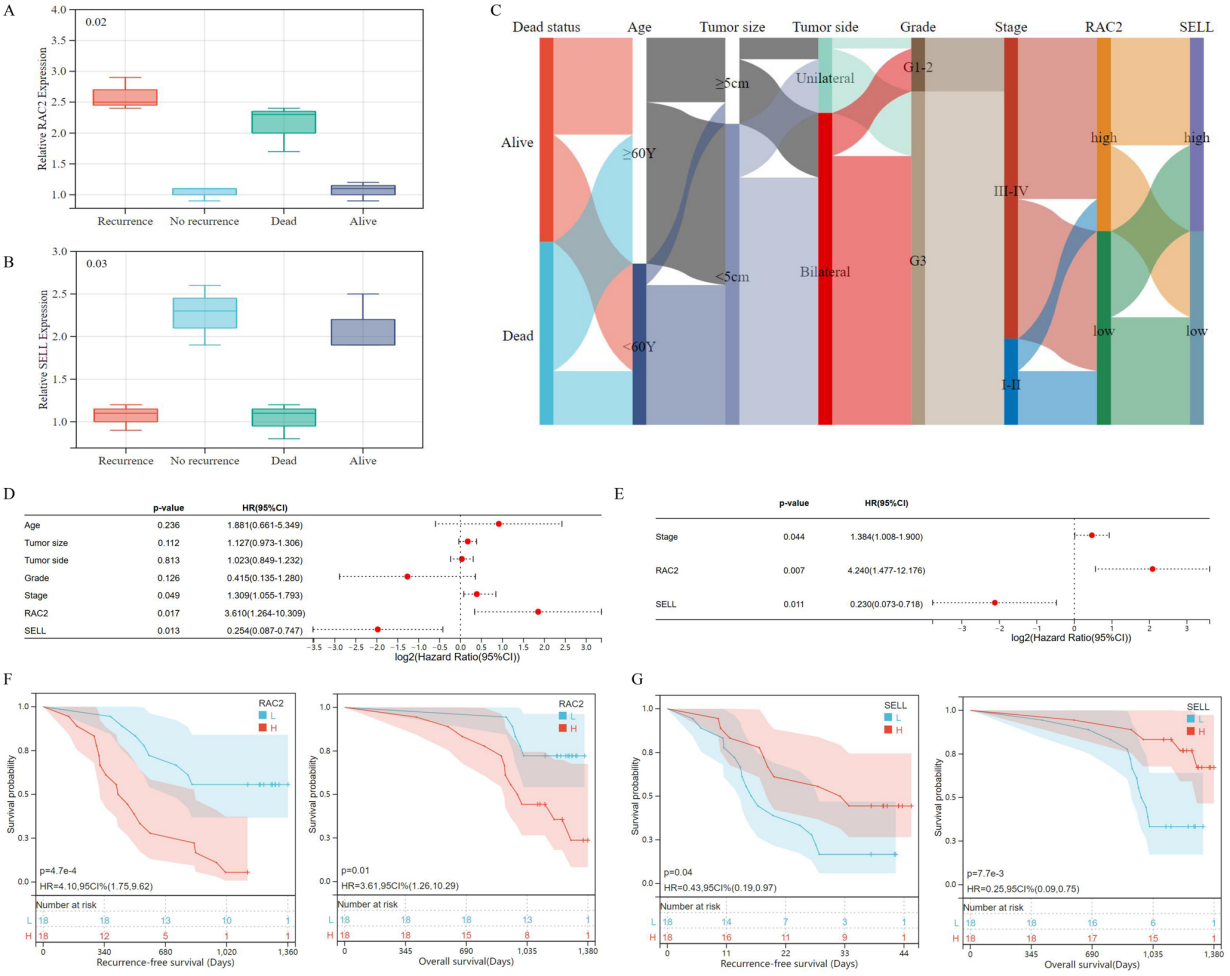
NETs‐related genes (RAC2 and SELL) could predict prognosis among OvCa individuals. Gene expression of RAC2 (A) and SELL (B) in OvCa tissues, measured through PCR analysis. (C) Sankey plot of NETs‐related signature and clinical characteristics, such as age, tumour size, tumour side, grade, clinical FIGO stage and survival status. Forest diagrams of (D) univariate and (E) multivariate Cox regression analysis for OvCa prognosis, based on clinical features and NRGs (RAC2 and SELL). The Kaplan–Meier survival curves for OvCa recurrence‐free survival (RFS, left) and overall survival (OS, right) were classified by expression of (F) RAC2 and (G) SELL.

**FIGURE 7:**
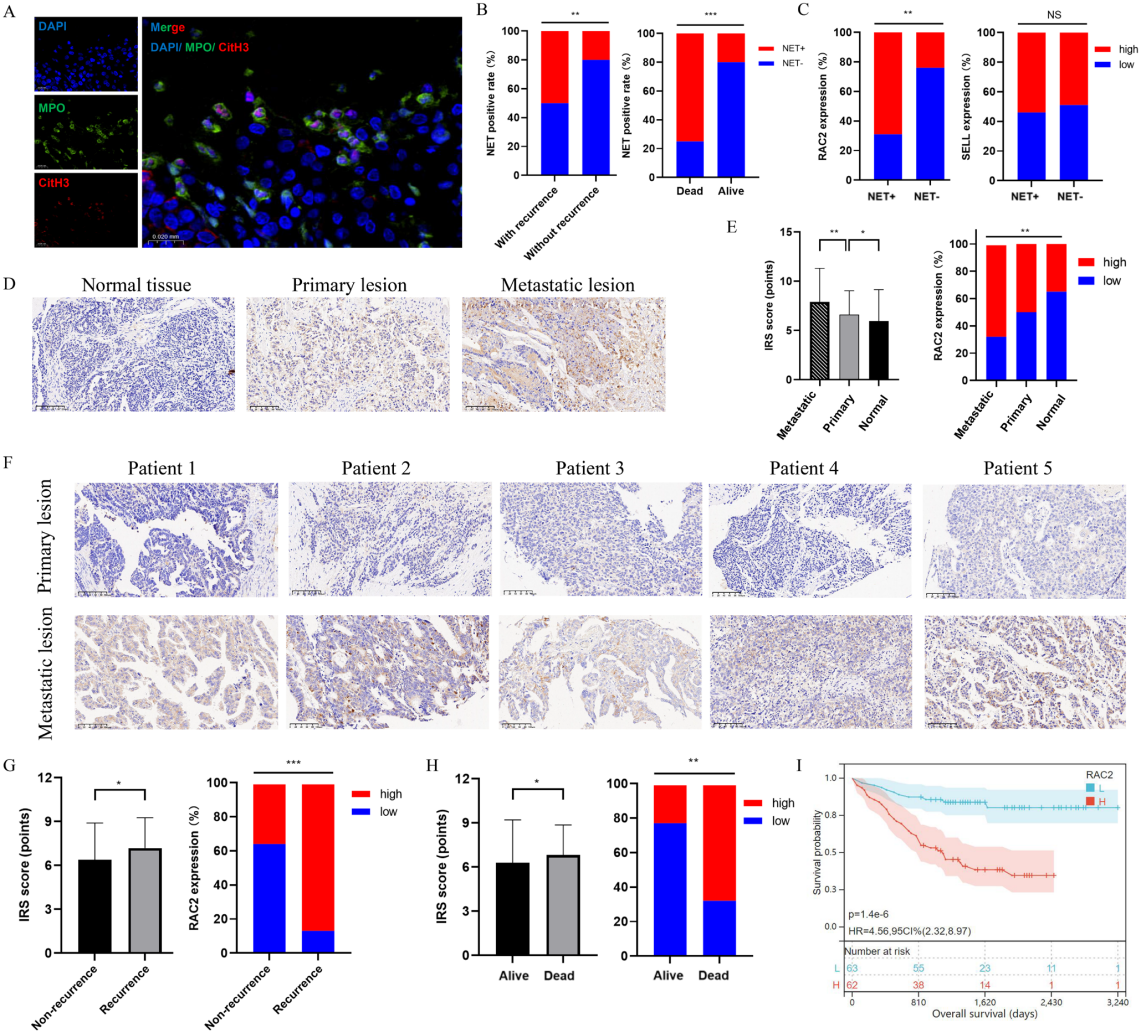
Aberrant upregulation of RAC2 related to NETs formation and tumour metastasis in ovarian cancer (OvCa). (A) Representative Multiplex immunohistochemical (mIHC) images of NETs, among which CitH3 (red) and MPO (green) were stained. Nuclei were stained with DAPI (blue). (B) OvCa patients with NETs were more likely to suffered recurrence (left) and death (right). (C) The relationship between NETs towards RAC2 (left) and SELL (left) expression among OvCa patients. (D) The immunohistochemistry (IHC) staining images of RAC2 expression in normal controls (left), primary OvCa tissues (middle) and metastatic OvCa tissues (right) were presented. (E) Metastatic OvCa tissues had significantly upregulated RAC2, compared with controls and primary tumour tissues, analysed through IRS score. (F) Representative IHC images staining RAC2 of primary (up) and metastatic OvCa lesions (down) of 5 OvCa patients. The RAC2 expression analysed by the IHC staining was over‐expressed among individuals suffered (G) recurrence or (H) death. (I) The Kaplan–Meier survival curves showed that RAC2 over‐expression was significantly associated with OvCa prognosis.

We included 36 OvCa individuals and followed them for 37.88 (31.48–42.72) months. Clinical features are listed in Table S3. We evaluated gene expression of RAC2 and SELL in OvCa tissues, through the qRT‐PCR analysis, which proved that OvCa patients with higher RAC2 (*p*‐value = 0.02, Figure 6A) and lower SELL expressions (*p*‐value = 0.03, Figure 6B) are more likely to suffer poor prognosis, which is consistent with the findings of bioinformatics analysis. In Figure 6C, we graphed the Sankey plot of the NETs‐related signature and clinical characteristics (age, tumour size, tumour side, grade, FIGO stage and survival status). We checked the proportional hazards assumption through cumulative risk function curves, which indicated that tumour size, tumour side and FIGO stage did not fit the proportional hazards assumption (Figure S3E–K). For those covariates don't fit the proportional hazards assumption, we applied the Time‐Dependent Cox Regression Model. Based on clinical features and NRGs, we conducted univariate (Figure 6D) and multivariate (Figure 6E) Cox analysis indicates that RAC2 and SELL (*p*‐value = 0.007 and 0.011) were prognostic indicators for OvCa, in addition to the clinical stage (*p*‐value = 0.044). The results showed that patients with high RAC2 and SELL expression were associated with a 4.240‐fold increase (95% CI 1.477–12.176) and 0.230‐fold increase (95% CI 0.073–0.718) of death rate, respectively, comparing to those with low expression. Meanwhile, patients with advance FIGO stage were associated with a 1.384‐fold increase (95% CI 1.008–1.900) of mortality, comparing to those with early clinical stage. Stepwise, we graphed K‐M survival curves, which proved that OvCa patients with higher RAC2 (Figure 6F) and lower SELL expressions (Figure 6G) suffered worse OS (*p*‐value < 0.05). We showed the representative Multiplex immunohistochemical (mIHC) images of NETs, which were defined by CitH3 (red) and MPO (green), while nuclei were stained with DAPI (blue, Figure 7A). Prognostic analysis identified that patients with NETs were more likely to suffer recurrence (Figure 7B, left) and tumour‐related death (Figure 7B, right, *p*‐value < 0.001). Through correlation analysis, we concluded that RAC2 upregulation was positively associated with NET formation (Figure 7C, left, *p*‐value < 0.01), while there was no significant relationship between SELL expression and NETs (Figure 7C, right). In Figure 7D, we applied the IHC analysis upon tissue microarrays containing OvCa patients (*n* = 125) and corresponding normal controls, which showed that RAC2 staining was located at cytosol and nucleus of OvCa cells. Figure 7E implied that metastatic OvCa lesions had significantly higher RAC2 expression (IRS score = 7.90 ± 3.37), compared with normal controls (IRS score = 5.95 ± 3.17) and primary tumour lesions (IRS score = 6.62 ± 2.41, *p*‐value < 0.05). Representatively, Figure 7F showed the IHC images staining RAC2 of primary (up) and metastatic OvCa lesions (down) from five OvCa individuals. We concluded that RAC2 was upregulated (*p*‐value < 0.05) among individuals who suffered (Figure 7G) recurrence or (Figure 7H) tumour‐related death. However, RAC2 expression showed no association with clinicopathological characteristics (*p*‐value > 0.01, Table S4). We also applied Bonferroni corrections to address potential false positives for multiple comparisons, which further proved that RAC2 expression had no significant association with patients with various clinicopathological features (including FIGO stage and Histology type). Subsequently, we applied Cox Regression analyses to define RAC2 expression (*p*‐value = 0.000) and clinical stage (*p*‐value = 0.008) as prognostic indicators for OvCa (Table S5), which showed that patients with high RAC2 expression were associated with a 4.001‐fold increase (95% CI 1.988–8.064) of OvCa death and advanced clinical stage (FIGO III‐IV) were associated with a 3.382‐fold increase (95% CI 1.371–8.344) of death rate. Next, we graphed K–M curves, which showed that RAC2 up‐expression was significantly associated with worse OS (*p*‐value < 0.001, Figure 7I). Collectively, aberrant upregulation of RAC2, one of the NETs‐related signature genes, was associated with OvCa metastasis and poor prognosis, probably by inducing NET formation.

## 4 Discussion

In this study, we comprehensively defined and validated a NETs‐related eight‐gene signature for prognosis prediction and drug sensitivity assessment among OvCa patients, based on bioinformatics analysis of public datasets. Especially, RAC2, one of the NETs‐related signature genes, was predominantly related to NET formation, thus providing hints towards the anti‐tumour response mechanism of NETs in OvCa.

A previous study by our team implied that neutrophils, the first defence line towards infection, could influence tumour progression in OvCa [24]. Recently, NETs have emerged as renewed forefront of neutrophil biology [25]. Recent evidence indicated that NETs, web‐like chromatin structures released in response to inflammatory cues, could trap and eliminate pathogens, thus playing a vital role in various inflammatory conditions, including autoimmune diseases and thrombosis [26]. With few exceptions, studies also reported the relationship between presence of NETs, drug sensitivity and prognosis in numerous malignancies including breast cancer [7], pancreatic cancer [8] and OvCa [27]. However, its vital role in cancer metastasis is still largely unknown. Recent research has shed light on the mechanisms of NETs in tumour metastasis, including remodelling of laminin to awaken dormant cancer cells and activate integrin inflammation signal [28], or altering tumour cell bioenergetics by releasing neutrophil elastase to induce PGC1α upregulation [29]. Moreover, Cools‐Lartigue et al. [30] reported that NETs could trap sequester circulating tumour cells, which were progenitors originated from primary tumour, entered bloodstream and invaded nearby tissues, thus promoting tumour metastasis in OvCa, supported by our previous research [31]. In the issue of immunity, Ireland and colleagues reported that NETs could form physical barrier between cancer cells and neighbouring cytotoxic immune cells (CD8+ T cells, NK cells, etc.) in tumour immune microenvironment to affect tumour progression and metastasis [26]. Consistently, in our study, we demonstrated that NET presence was associated with survival via bioinformation analysis of TCGA‐OvCa and ICGC‐OvCa cohorts, as well as IHC analysis for tissue microarrays of 125 OvCa patients.

To date, though several studies reported the relationship between NETs and tumour metastasis, no NETs‐related gene signature has been applied from brench to bedside, might because of limitations in sensitivity and specificity of the signature identified. In lung cancer, Fang et al. [32] built a 12‐gene NETs‐related lncRNA signature, which had a superior trend towards prognosis in TCGA training and validation cohorts (*p*‐value < 0.0001 and *p*‐value = 0.0023). Another study by Li defined seven‐gene NET‐related signature, including ITGA5, NIFK, NUTF2, PDGFα, TNFRSF12, LINC00460 and LINC02454, for head and neck squamous cell carcinoma through the TCGA and ICGC cohorts (*p*‐value < 0.05) [33]. However, as for OvCa, no study has constructed a NETs‐related prognostic signature till now, partly due to its ‘cold’ immune microenvironment [9]. Thus, we tried to define a promising eight‐gene NET‐related signature (including ELN, FBN1, IL1B, LCN2, MMP2, MMP9, RAC2 and SELL) from 87 NRGs filtered in Genecards dataset through the LASSO‐COX analysis. The prognostic value of the signature was validated in TCGA‐OvCa and ICGC‐OvCa cohorts. To our knowledge, we are initial to develop this NETs‐related signature, which could be a reliable tool for prognosis prediction and drug sensitivity assessment among OvCa patients.

Among the eight identified signature genes, the mechanisms of RAC2 and SELL haven't been explored yet in OvCa. SELL, a cell surface adhesion molecule belonging to the adhesion receptor family, could facilitate leucocytes to bind and roll upon endotheliocytes, thus leading to migration in inflammation sites [34]. Watson et al. reported that SELL upregulation in tumour‐specific T cells could offer advantage of cancer immunotherapy, which might benefit clinical applications of modifying CAR‐T cancer therapy in mouse models [22]. The RAS‐related C3 botulinum toxin substrate 2, also known as RAC2, is associated with immune function, as a vital member of RHO subclass of RAS superfamily GTPases [35]. For instance, Arrington et al. [36] found that the E62K mutant of GTPase RAC2 could induce immune function disorder by enhancing RAC2 hyperactivation, changing guanine nucleotide exchange factor specificity and impairing GTPase‐activating protein function. Nowadays, as for RAC2, an important molecular of NETs, there have been no studies focused on RAC2 in OvCa till now. Thus, we tried to fill up the blank and evaluate the role of RAC2 in OvCa metastasis. Especially, we initially claimed that RAC2 was predominantly related to NET formation and metastasis in OvCa through both bioinformation analyses of the TCGA dataset and IHC analysis of the tissue microarrays, though definite mechanism needs further exploration.

Nowadays, many findings support cross‐talk between tumour and immune cells in microenvironment, which contributes to tumour metastasis [23]. However, the mechanisms of how NETs influence neighbouring cells and regulate immune response remain unclear [5]. Accordingly, we conducted CIBERSORT analysis to estimate the composition of 22 immune cells in OvCa tissues of TCGA‐OvCa individuals divided by NETs‐related signature. The results implied that resting memory CD4+ T cells, follicular helper T cells and neutrophils were significantly upregulated among high‐risk individuals, while macrophage and activated memory CD4+ T cells were upregulated among low‐risk individuals. Consistently, Zhang et al. [37] found that OvCa tumour cells could differentiate M0 into M2 macrophages in microenvironment through regulating ERK signalling pathway that could augment cancer migration. As for T cells, Kaltenmeier et al. [38] claimed that in the NET‐rich tumour microenvironment, majority of both CD4+ T cells and CD8+ T cells over‐expressed inhibitory receptors and showed functional exhausted phenotype. Wang et al. [39] also concluded that NETs could bridge innate and adaptive immunity by promoting Regulatory T cells (Tregs) differentiation through metabolic reprogramming of CD4+ T cells, thus contributing to carcinogenesis.

However, our study still has several limitations. Firstly, molecular mechanisms of NETs‐related signature in OvCa are needed to explore using cell lines and animal models. Moreover, our NET‐related signatures were based only on the TCGA database and validated on the ICGC database, which might have selection bias. Further multicentre large cohorts are still needed to validate the signature in clinics.

## 5 Conclusion

Conclusively, our study comprehensively assessed the vital role of NETs in OvCa and developed an eight‐gene prognostic signature (including ELN, FBN1, IL1B, LCN2, MMP2, MMP9, RAC2 and SELL) via bioinformatics algorithms. We also validated that the NETs‐related prognostic signature was a reliable tool for drug sensitivity prediction and immune landscape assessment among OvCa patients, thus assisting decision‐making in OvCa. Especially, we initially identified the importance of RAC2, one of the NETs‐related signature genes, in OvCa metastasis by inducing NETs formation, which could provide hints towards the anti‐tumour response of NETs, though definite mechanism needs further exploration.

## Author Contributions

**Yue Zhang:** conceptualization (equal), data curation (equal), writing – original draft (equal). **Chao Wang:** conceptualization (equal), data curation (equal), writing – original draft (equal). **Shanshan Cheng:** formal analysis (equal), investigation (equal), methodology (equal). **Yanna Xu:** methodology (equal), project administration (equal). **Sijia Gu:** resources (equal), software (equal). **Yaqian Zhao:** supervision (equal), validation (equal). **Jiani Yang:** funding acquisition (equal), validation (equal), visualization (equal), writing – review and editing (equal). **Yu Wang:** supervision (equal), validation (equal), visualization (equal), writing – review and editing (equal).

## Ethics Statement

All patients involved provided informed consents for the usage of information for research purposes. The study was approved by the Ethics Committee of the Renji Hospital Affiliated to Shanghai Jiaotong University School of Medicine.

## Consent

All the authors approved the submission to this journal and the final version of the manuscript.

## Conflicts of Interest

The authors declare no conflicts of interest.

## Supporting information


Appendix S1:

## Data Availability

The data and materials that support the findings are available from corresponding authors upon reasonable request.
