# Supplementary material for: A Neutrophil Extracellular Traps–Related Signature Predicts Clinical Outcomes and Identifies Immune Landscape in Ovarian Cancer
Source: J Cell Mol Med. 2024 Dec 27;28(24):e70302. doi: 10.1111/jcmm.70302 (PMC11680186; doi:10.1111/jcmm.70302)
Supplement: Supplementary file 1 — Appendix S1: [file JCMM-28-e70302-s001.zip › Supplement figure 2.docx]

**Supplement Figure 2. Validation** **of** **NETs-related prognostic signature in OvCa.** Distribution (top) and scatter diagrams (middle) showed NETs-related riskscore of OvCa individuals, according to survival time and status, in the (A) TCGA-OvCa training and (B) ICGC-OvCa validation cohorts. Heatmaps (bottom) represented expression of 8 NETs-related genes, including ELN, FBN1, IL1B, LCN2, MMP2, MMP9, RAC2, and SELL, between 2 risk groups. The Kaplan-Meier curves for patients OS in the (C) TCGA-OvCa training and (D) ICGC-OvCa validation cohorts, who were classified into 2 groups by the NETs-related prognostic signature. The Receiver Operating Characteristic curve analysis of the signature in the (E) TCGA-OvCa training and (F) ICGC-OvCa validation cohorts.

**
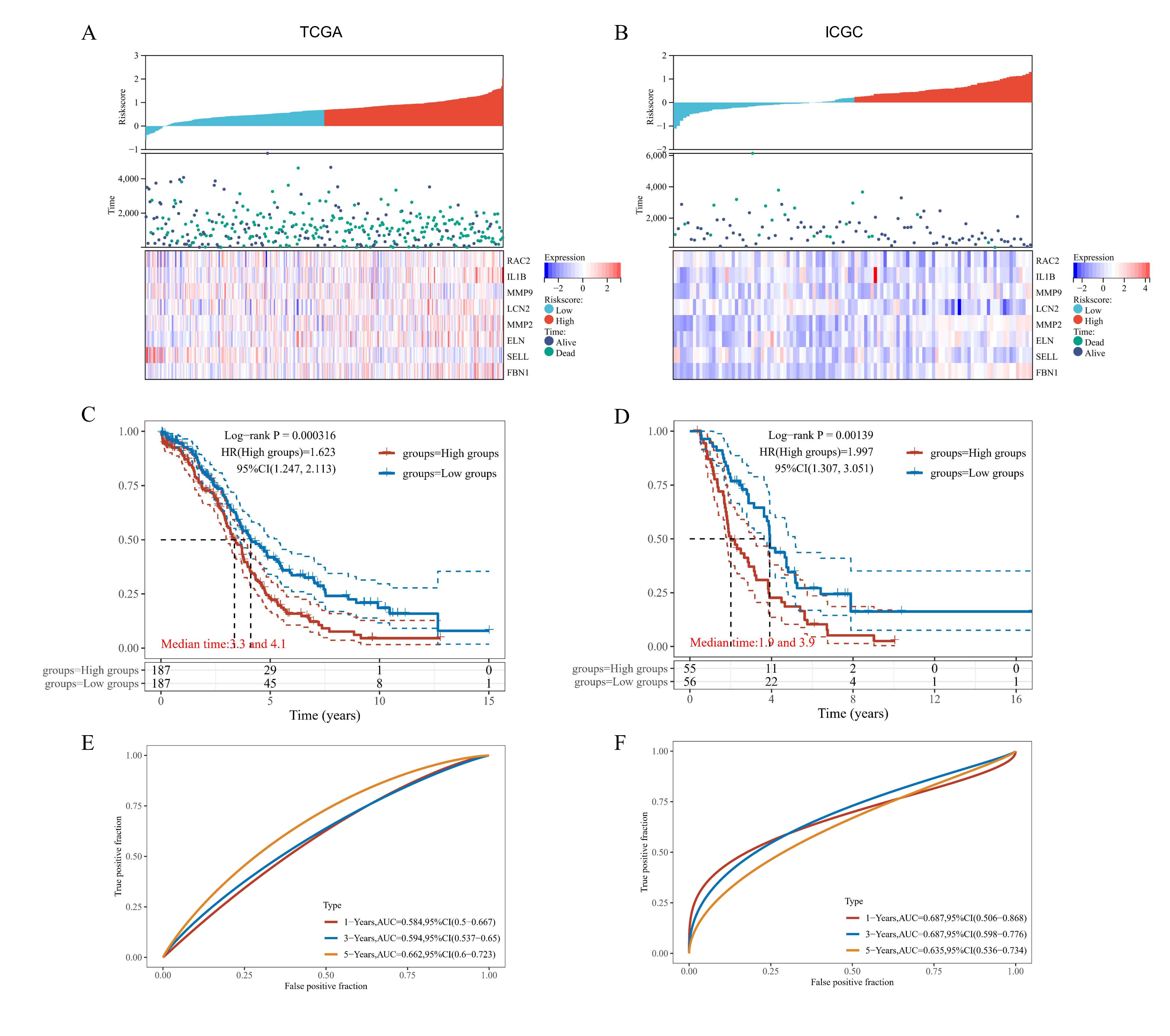
**
