# Supplementary material for: A Neutrophil Extracellular Traps–Related Signature Predicts Clinical Outcomes and Identifies Immune Landscape in Ovarian Cancer
Source: J Cell Mol Med. 2024 Dec 27;28(24):e70302. doi: 10.1111/jcmm.70302 (PMC11680186; doi:10.1111/jcmm.70302)

**Supplement figure 3.** Check the proportional hazards assumption for Cox regression analysis of clinical features, such as (A) age, (B) clinical stage, and (C) pathological grade, and (D) NETs-related signature riskscore, among the TCGA-OvCa cohort. Check the proportional hazards assumption for Cox regression analysis of clinical features, such as (E) age, (F) tumor size, (G) tumor side, (H) grade, (I) clinical FIGO stage, and NRGs, including (J) RAC2 and (K) SELL among OvCa patients at our institution.


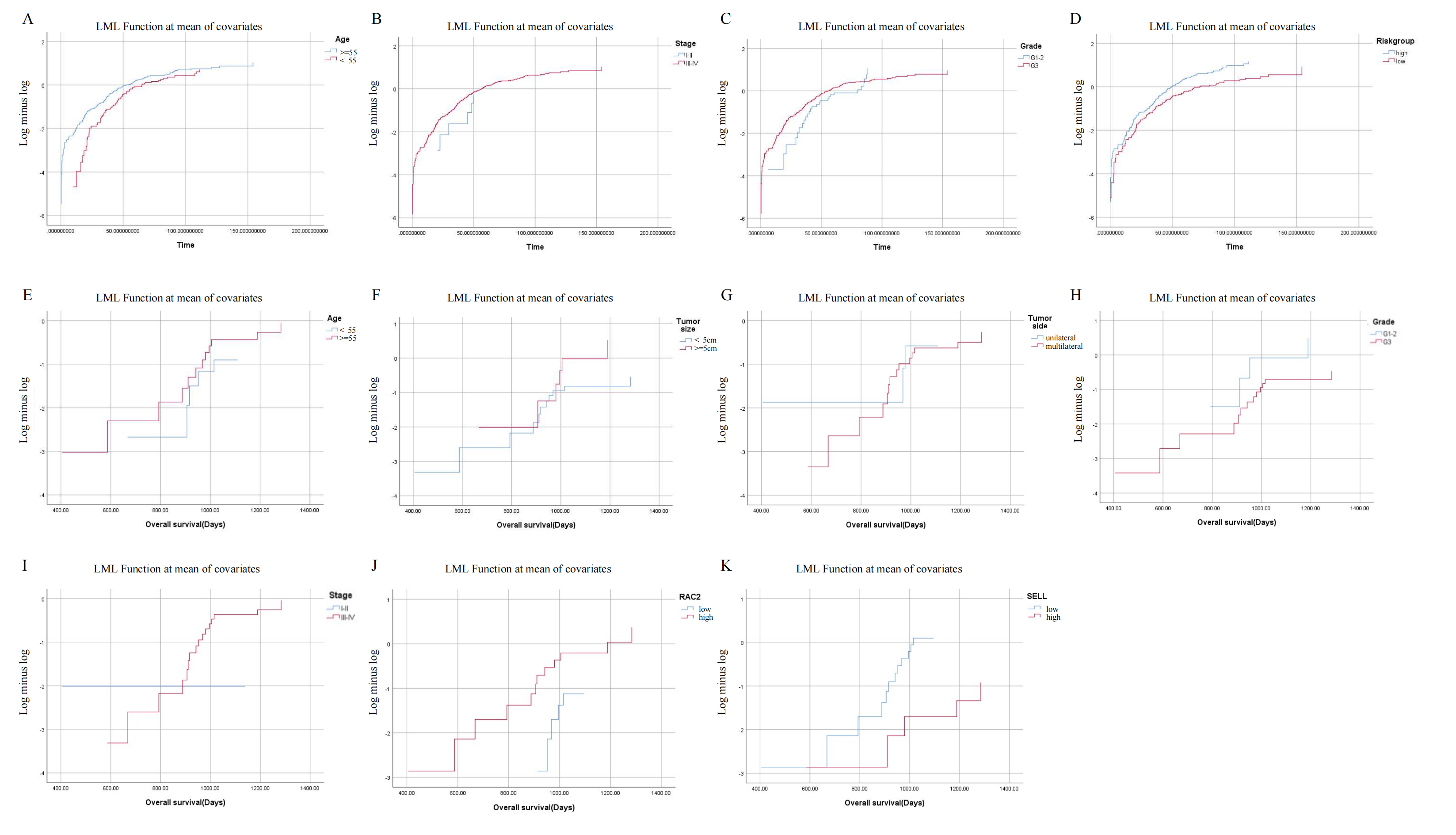

Supplement: Supplementary file 1 — Appendix S1: [file JCMM-28-e70302-s001.zip › Supplement figure 3.docx]
