# Supplementary material for: A Neutrophil Extracellular Traps–Related Signature Predicts Clinical Outcomes and Identifies Immune Landscape in Ovarian Cancer
Source: J Cell Mol Med. 2024 Dec 27;28(24):e70302. doi: 10.1111/jcmm.70302 (PMC11680186; doi:10.1111/jcmm.70302)
Supplement: Supplementary file 1 — Appendix S1: [file JCMM-28-e70302-s001.zip › Supplement figure 4.docx]

**Supplement Figure 4. Single-cell analysis and somatic alteration landscape of the NETs pattern.** (A) The Uniform Manifold Approximation and Projection diagram graohed high-quality cells of 10× Genomics single-cell transcriptome expression from 5 OvCa patients and 2 normal controls, published by Qian and colleagues in the GEO database ^10^. (B) Pseudo-time trajectory analysis in OvCa tissues of 10 cell-types identified by specific markers. Pseudo-time trajectory analysis in OvCa tissues of 10 cell-types with (C) RAC2 and (D) SELL expression. (E) Pseudo-time trajectory analysis in OvCa tissues of Myeloid cells. Pseudo-time trajectory analysis in OvCa tissues of Myeloid cells with (F) RAC2 and (G) SELL expression. (H) The genomic aberrations landscape of TCGA- OvCa individuals, divided by the NETs-related signature. The top 20 frequency of gene alterations were listed.

**
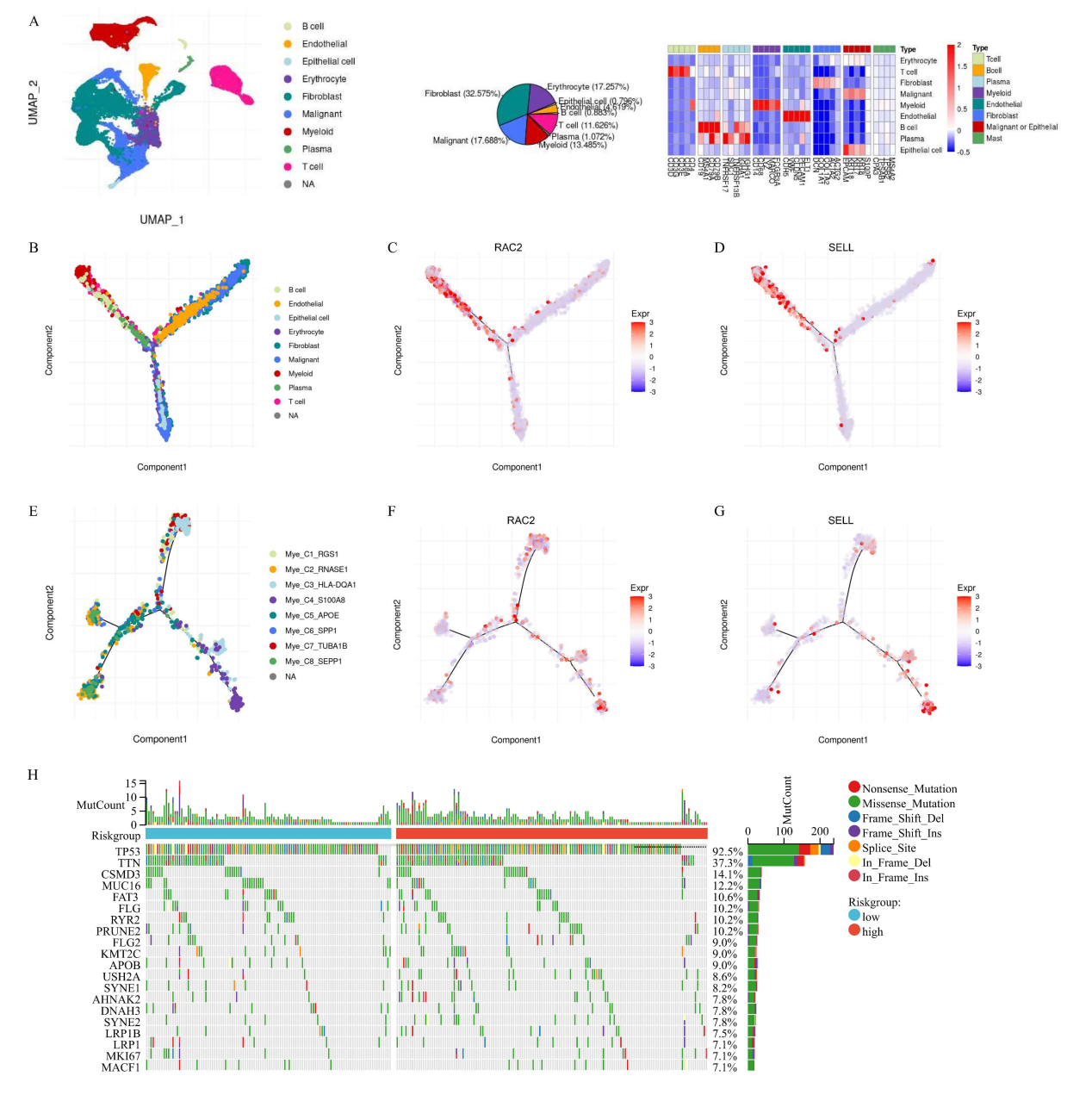
**
