# Supplementary material for: A Neutrophil Extracellular Traps–Related Signature Predicts Clinical Outcomes and Identifies Immune Landscape in Ovarian Cancer
Source: J Cell Mol Med. 2024 Dec 27;28(24):e70302. doi: 10.1111/jcmm.70302 (PMC11680186; doi:10.1111/jcmm.70302)
Supplement: Supplementary file 1 — Appendix S1: [file JCMM-28-e70302-s001.zip › Supplement figure 6.docx]

**Supplement Figure 6. Pan-cancer analysis of the NETs-related signature.** Radar charts showed expression of (A) RAC2 and (B) SELL in pan-cancer. (C) Association between the immune landscape and NETs-related riskscore in pan-cancer, which was analyzed via the CIBERSORT algorithm.
