# Supplementary material for: A Neutrophil Extracellular Traps–Related Signature Predicts Clinical Outcomes and Identifies Immune Landscape in Ovarian Cancer
Source: J Cell Mol Med. 2024 Dec 27;28(24):e70302. doi: 10.1111/jcmm.70302 (PMC11680186; doi:10.1111/jcmm.70302)
Supplement: Supplementary file 1 — Appendix S1: [file JCMM-28-e70302-s001.zip › Supplement table 1.docx]

**Supplement Table 1: The overview of 8 Neutrophil Extracellular Traps (NETs)-related signature genes.** ^13-22^

| **Symbol** | **Gene Name** | **Summary of the function in ovarian cancer (OvCa)** | **Refer** |
| --- | --- | --- | --- |
| ELN | Elastin | ELN could encode elastic fibers, which comprise extracellular matrix and confer elasticity to tissues. Elastin peptides could modulate the cellular physiology of endothelial cells, tumor cells, stromal fibroblasts, and inflammatory cells in cancer, including OvCa. | ^13^ |
| FBN1 | Fibrillin 1 | FBN1 induces chemoresistance by modulating glycolysis and angiogenesis through the VEGFR2/STAT2 signaling axis in OvCa cells. | ^14^ |
| IL-1β | Interleukin 1 Beta | IL-1β, a member of the interleukin 1 cytokine family, is produced by activated macrophages as an important mediator of inflammatory response. In OvCa, the IL-1β/β1-integrin axis could promote tumor dissemination by modulating cell adhesion to mesothelium. | ^15,16^ |
| LCN2 | Lipocalin 2 | LCN2, a vital regulator of tumorigenesis, could promote OvCa proliferation and migration by activating the ERK/GSK3β/β-catenin signaling axis. | ^17^ |
| MMP2 | Matrix Metallopeptidase 2 | MMP2 is a zinc-dependent enzyme capable of cleaving signal transduction molecules and extracellular matrix components. In OvCa, MKL1 could induce OvCa migration and invasion by promoting MMP2 transcription. | ^18^ |
| MMP9 | Matrix Metallopeptidase 9 | MMP9, a vital member of the matrix metalloproteinase family, could control lactate production, OvCa metastasis, and stemness regulation via the NANOG/SOX9 signaling axis. | ^19^ |
| RAC2 | Rac Family Small GTPase 2 | RAC2, exclusively expressed in blood-derived cells, has key roles in actin-based cytoskeleton formation and cell signaling regulation. RAC2 could induce abnormal proliferation of quiescent cells in non-small cell carcinoma by promoting JUNB expression through the MAL-SRF signaling axis. Unknow in OvCa. | ^20,21^ |
| SELL | Selectin L | SELL, as a common T cell activation marker, could regulate naïve T cell recruitment from the bloodstream and benefit T cell selection in adoptive cell therapy for solid tumors. Unknow in OvCa. | ^22^ |
