# Supplementary material for: A Neutrophil Extracellular Traps–Related Signature Predicts Clinical Outcomes and Identifies Immune Landscape in Ovarian Cancer
Source: J Cell Mol Med. 2024 Dec 27;28(24):e70302. doi: 10.1111/jcmm.70302 (PMC11680186; doi:10.1111/jcmm.70302)
Supplement: Supplementary file 1 — Appendix S1: [file JCMM-28-e70302-s001.zip › Supplement table 2.docx]

**Supplement Table 2.** **The correlation between NETs-related riskscore and clinical features of TCGA-OvCa patients.**

| **Characteristic** | **No. of patients** | **NETs-related riskscore** | | **P-value** |
| --- | --- | --- | --- | --- |
|  |  | **Low** | **High** |  |
| **Age (n,%)** |  |  |  | 0.175 |
| **<55 years** | 129(35.6%) | 53(14.6%) | 76(21.0%) | - |
| **≥55 years** | 233(64.4%) | 113(31.2%) | 120(33.1%) | - |
| **FIGO stage (n,%)** |  |  |  | 0.015 |
| **I-II** | 21(5.8%) | 15(4.1%) | 6(1.7%) | - |
| **III-IV** | 341(94.2%) | 151(41.7%) | 190(52.5%) | - |
| **Pathology grade (n,%)** |  |  |  | 0.927 |
| **I-II** | 43(11.9%) | 20(5.5%) | 23(6.4%) | - |
| **III** | 319(88.1%) | 146(40.3%) | 173(47.8%) | - |

Abbreviation: FIGO stage, Federation International of Gynecology and Obstetrics stage
