# Supplementary material for: A Neutrophil Extracellular Traps–Related Signature Predicts Clinical Outcomes and Identifies Immune Landscape in Ovarian Cancer
Source: J Cell Mol Med. 2024 Dec 27;28(24):e70302. doi: 10.1111/jcmm.70302 (PMC11680186; doi:10.1111/jcmm.70302)
Supplement: Supplementary file 1 — Appendix S1: [file JCMM-28-e70302-s001.zip › Supplement table 3.docx]

**Supplement table 3. The baseline characteristics of ovarian cancer (OV) patients involved in analysis.**

| **Features** | **Number of cases (n, %)** |
| --- | --- |
| Age |  |
| <60 years | 15 (41.67%) |
| ≥60 years | 21 (58.33%) |
| FIGO stage |  |
| I-II | 8 (22.22%) |
| III-IV | 28 (77.78%) |
| Pathological grade |  |
| G1-2 | 5 (13.89%) |
| G3 | 31 (86.11%) |
| Tumor size |  |
| <6 cm | 17 (47.22%) |
| ≥6 cm | 19 (52.78%) |
| Ascites |  |
| <1000ml | 18 (50.00%) |
| ≥1000ml | 18 (50.00%) |
| Survival status |  |
| Alive | 19 (52.78%) |
| Dead | 17 (47.22%) |
