# Supplementary material for: A Neutrophil Extracellular Traps–Related Signature Predicts Clinical Outcomes and Identifies Immune Landscape in Ovarian Cancer
Source: J Cell Mol Med. 2024 Dec 27;28(24):e70302. doi: 10.1111/jcmm.70302 (PMC11680186; doi:10.1111/jcmm.70302)
Supplement: Supplementary file 1 — Appendix S1: [file JCMM-28-e70302-s001.zip › Supplement table 4.docx]

**Supplement Table 4.** **The correlation between RAC2 expression and clinical features of 125 ovarian cancer (OvCa) patients.**

| **Characteristic** | **No. of patients** | **RAC2 expression** | | **P-value** |
| --- | --- | --- | --- | --- |
|  |  | **Low**  **(IRS score<8)** | **High**  **(IRS score≥8)** |  |
| **Age (n,%)** |  |  |  | 0.073 |
| **<55 years** | 56(44.8%) | 33(26.4%) | 23(18.4%) | - |
| **≥55 years** | 69(55.2%) | 29(23.2%) | 40(32.0%) | - |
| **FIGO stage (n,%)** |  |  |  | 0.016 |
| **I** | 31(24.8%) | 10(8.0%) | 21(16.8%) | - |
| **II** | 14(11.2%) | 5(4.0%) | 9(7.2%) |  |
| **III** | 60(48.0%） | 32(25.6%) | 28(22.4%) | - |
| **IV** | 20(12.0%） | 15(12.0%) | 5(4.0%) | - |
| **Pathology grade (n,%)** |  |  |  | 0.281 |
| **I-II** | 54(43.2%) | 30(24.0%) | 24(19.2%) | - |
| **III** | 71(56.8%) | 32(25.6%) | 39(31.2%) | - |
| **Histology type (n,%)** |  |  |  | 0.729 |
| **Serous** | 78(62.4%) | 41(32.8%) | 37(29.6%) | - |
| **Mucous** | 11(8.8%) | 6(4.8%) | 5(4.0%) | - |
| **Endometrioid** | 14(11.2%) | 6(4.8%) | 8(6.4%) | - |
| **Other types** | 22(17.6%) | 9(7.2%) | 13(10.4%) | - |
| **Tumor diameter (n,%)** |  |  |  | 0.593 |
| **<10 cm** | 62(49.6%) | 29(23.2%) | 33(26.4%) | - |
| **≥10 cm** | 63(50.4%) | 33(26.4%) | 30(24.0%) | - |
| **Serum CA125 (n, %)** |  |  |  | 0.808 |
| **<35 U/ml** | 20(16.0%) | 9(7.2%) | 11(8.8%) | - |
| **≥35 U/ml** | 105(84.0%) | 53(42.4%) | 52(41.6%) | - |

Abbreviation: FIGO stage, Federation International of Gynecology and Obstetrics stage
