# Supplementary material for: A Neutrophil Extracellular Traps–Related Signature Predicts Clinical Outcomes and Identifies Immune Landscape in Ovarian Cancer
Source: J Cell Mol Med. 2024 Dec 27;28(24):e70302. doi: 10.1111/jcmm.70302 (PMC11680186; doi:10.1111/jcmm.70302)
Supplement: Supplementary file 1 — Appendix S1: [file JCMM-28-e70302-s001.zip › Supplement table 5.docx]

**Supplement Table 5. Univariate and multivariate Cox Regression analysis of prognostic indicators among 125 ovarian cancer (OvCa) patients.**

| **Characteristic** | **Univariate Analysis** | | **Multivariate Analysis** | |
| --- | --- | --- | --- | --- |
|  | **HR (****95% CI)** | **P-value** | **HR (95% CI)** | **P-value** |
| **Age** |  |  |  |  |
| **<55 years** | Reference | - | Reference | - |
| **≥55 years** | 1.052(0.595-1.862) | 0.861 | 1.123(0.621-2.032) | 0.702 |
| **FIGO stage** |  |  |  |  |
| **I-II** | Reference | - | Reference | - |
| **III-IV** | 4.238(1.897-9.471) | 0.001 | 3.382(1.371-8.344) | 0.008 |
| **Pathology grade** |  |  |  |  |
| **I-II** | Reference | - | Reference | - |
| **III** | 0.727(0.413-1.282) | 0.271 | 0.825(0.434-1.569) | 0.558 |
| **Histology type** |  | 0.850 |  | 0.631 |
| **Serous** | Reference | - | Reference | - |
| **Mucous** | 0.591(0.181-1.932) | 0.384 | 1.239(0.302-5.077) | 0.766 |
| **Endometrioid** | 0.875(0.341-2.245) | 0.780 | 1.395(0.528-3.682) | 0.502 |
| **Other types** | 0.977(0.450-2.122) | 0.953 | 1.737(0.718-4.200) | 0.220 |
| **Tumor diameter** |  |  |  |  |
| **<10 cm** | Reference | - | Reference | - |
| **≥10 cm** | 1.253(0.710-2.211) | 0.437 | 1.373(0.756-2.493) | 0.298 |
| **Serum CA125** |  |  |  |  |
| **<35 U/ml** | Reference | - | Reference | - |
| **≥35 U/ml** | 2.680(0.960-7.486) | 0.060 | 1.777(0.561-5.630) | 0.329 |
| **RAC2 expression** |  |  |  |  |
| **Low (IRS score<8)** | Reference | - | Reference | - |
| **High (IRS score≥8)** | 4.525(2.298-8.849) | 0.000 | 4.001(1.988-8.064) | 0.000 |

Abbreviation: HR, hazard ratio; 95% CI, 95% confidence interval; FIGO stage, Federation of International of Gynecologists and Obstetricians stage
